# Assessing gut barrier integrity and reproductive performance following pre-mating oral administration of solid-lipid-nanoparticles designed for drug delivery

**DOI:** 10.3389/ftox.2024.1508598

**Published:** 2025-01-07

**Authors:** Valentina Lacconi, Micol Massimiani, Giulia Antonello, Paolo Gasco, Roberta Bernardini, Cristiana Ferrari, Lorenzo Ippoliti, Gina La Sala, Antonio Pietroiusti, Ivana Fenoglio, Chiara Riganti, Luisa Campagnolo

**Affiliations:** ^1^ Department of Biomedicine and Prevention, University of Rome Tor Vergata, Rome, Italy; ^2^ Departmental Faculty of Medicine, Saint Camillus International University of Health Sciences, Rome, Italy; ^3^ Department of Chemistry, University of Torino, Torino, Italy; ^4^ Nanovector S.r.l., Torino, Italy; ^5^ Department of Translational Medicine and Centro Interdipartimentale di Medicina Comparata, Tecniche Alternative ed Acquacoltura (CIMETA), University of Rome “Tor Vergata”, Rome, Italy; ^6^ Department of Occupational Medicine, University of Rome Tor Vergata, Rome, Italy; ^7^ Institute of Biochemistry and Cell Biology, Italian National Research Council (CNR), Monterotondo Scalo, Italy; ^8^ Department of Oncology, University of Torino, Torino, Italy

**Keywords:** solid-lipid-nanoparticles, pregnancy, gut barrier integrity, placenta, embryo, oral administration, fertility

## Abstract

Solid lipid nanoparticles (SLNs) have gained interest as drug delivery carriers due to their efficient cellular internalization and increased therapeutic effect of the loaded drug, with minimal side effects. Although recently several studies have shown the possibility to administer SLNs during pregnancy to vehicle mRNA to the placenta, data about the effect of premating exposure to SLNs on pregnancy outcome are scant. Considering that assumption of drug-delivering nanocarriers in reproductive age may potentially affect women’s reproductive health, the aim of the present study was to evaluate whether repeated oral administration of SLNs to female mice prior to mating would influence key pregnancy outcomes. For this purpose, SLNs melatonin loaded (SLN + mlt) or unloaded were orally administered to CD1 female mice at two different dosages—low (7.5 mg/kg) and high (750 mg/kg) —three times a week for 6 weeks. Females mice were mated and pregnancy was monitored from conception to delivery. All the assessed pregnancy parameters, including time to pregnancy, pregnancy duration, litter size, and the presence of any gross anomalies in the pups, and maternal key biochemical parameters were not significantly affected by SLN administration. Embryonic development was also evaluated and no effects on the number of implantation sites, fetus numbers, incidence of fetal resorptions, and measurements of crown-rump length, as well as fetal and placental weights, were observed in the treated mothers. The impact of SLNs on maternal intestinal barrier integrity and inflammation was assessed both *in vivo* in mice and *in vitro* using an intestinal epithelial barrier model by qRT-PCR. Results showed that unloaded SLNs, but not the SLN + mlt, affected intestinal barrier integrity. Although variation in the expression of inflammatory cytokines was recorded, this did not reflect in significant histological alterations and the integrity of the intestinal barrier was maintained. The *in vitro* model further confirmed the biocompatibility of SLNs, showing that both loaded and unloaded SLNs did not affect the integrity of the simulated intestinal epithelial barrier. In conclusion, these data suggest that administering SLNs, as a drug delivery vehicle, prior to conception does not affect either maternal health or fetal development, posing no risk to future pregnancy.

## 1 Introduction

The increased application of nanomaterials (NMs) and nanotechnology in many consumer products as well as in several biomedical applications has resulted in an heighted exposure of humans and the environment (Pietroiusti et al., 2018; Pietroiusti et al., 2017). NMs offer great promise in a wide range of disciplines such as chemistry, physics, biology, medicine and engineering thank to their unique properties, different from those of their bulk counterparts. Over the last decades, their toxicological properties and long-term impact on human health have been only partly elucidated and further extensive targeted studies are highly warranted (Bleeker et al., 2013).

A toxicological and safety evaluation of NMs appears even more relevant when the potential of nanotechnology is applied to nanomedicine. This is particularly true for applications of nanoparticles for drug delivery, where the particles are intentionally introduced into the human body, requiring precise toxicity assessment to ensure a safe use for humans, especially those individuals which may present increased vulnerability (De Jong and Borm, 2008; Aillon et al., 2009; Papp et al., 2008). Indeed, human exposure to NMs may be of particular concern for vulnerable populations, such as pregnant women and the developing fetus. Published studies have shown that NMs can cross the placental barrier of pregnant mice causing neurotoxicity of their offspring, and data on the ability of NMs to cross the human placental barrier have been provided by *ex vivo* experiments. However, indirect effects on fetal development due to NM placental accumulation have been also suggested (Yamashita et al., 2011; Sood et al., 2011).

Human exposure to NMs occurs mainly through inhalation (Malakar et al., 2021); however, oral ingestion is another relevant route of exposure, particularly when NMs are designed as potential drug carriers to efficiently increase absorption of specific medications. Available data on the toxicokinetic of orally administered NMs suggest that uptake and absorption of NMs in the gastrointestinal tract (GIT) may have major implications, with local and systemic effects (Pietroiusti et al., 2017).

The most widely used NMs for drug delivery in the biomedical field include lipid-based nanoparticles (LNPs), solid lipid nanoparticles (SLNs), nanosuspensions, nanoemulsions, and nanocrystals (Lingayat et al., 2017). Among these, SLNs are particularly useful for encapsulating drugs with limited intracellular accessibility, thus enhancing their bioavailability. SLNs are composed of spherical particles with a solid lipid core that can carry the drug, typically ranging in diameter from about 50 to 1,000 nm (Müller et al., 2000; Mukherjee et al., 2009). The use of SLNs for drug delivery offers an innovative approach with multiple advantages, such as facilitating cellular uptake and enhancing the therapeutic effect of the encapsulated drug, while minimizing potential side effects (Madkhali, 2022).

Data from the literature highlight several advantages for using SLNs as drug carriers: they are made from physiological compounds, do not require organic solvents for preparation, enable rapid large-scale production, and enhance drug stability. Additionally, SLNs are versatile in various applications, including active targeting. When surface-modified with specific molecules or ligands, SLNs can be directed to bind to specific cells or tissues, thereby facilitating selective drug release to target organs (Mehnert and Mäder, 2001; Bunjes, 2011). For instance, encapsulating antibiotics in SLNs improves their delivery to internal targets by enhancing intestinal absorption and bypassing efflux pumps, thus improving therapeutic efficacy (Hosseini et al., 2019; Ghaffari et al., 2010). Moreover, SLNs are capable of delivering both hydrophilic and lipophilic anticancer agents, improving drug penetration into cancer cells, potentially overcoming resistance, and minimizing adverse effects compared to unencapsulated drugs (Qureshi et al., 2017; Oliveira et al., 2016; Kang et al., 2010). Given the critical role of oral exposure in NM pharmacokinetics, particularly the absorption of drugs and SLNs in the ileum and jejunum (Qi et al., 2020; Alqahtani et al., 2021; Xu et al., 2021), gastrointestinal (GI) conditions during digestion should be considered. Indeed, variations in temperature, pH, salt concentrations, and enzyme activity can alter NM properties, including surface chemistry, agglomeration, and biomolecule interactions, potentially influencing toxicity and efficacy (Soliman et al., 2024; Boisen and Eggum, 1991). To our knowledge, no studies have so far reported on the potential toxicity of premating administration of SLNs on pregnancy outcome. However, data are available on the safety of a different type of LNPs administered during pregnancy. Indeed, two different groups have evaluated the possibility to use the lipid nanoparticles to deliver mRNA to the placenta. In 2022, Young et al. tested different types of LNPs to deliver Placental Growth Factor mRNA to the trophoblast both *in vitro* and in pregnant mice. They demonstrated that a specific combination of ionizable lipids and phospholipids achieved optimal mRNA delivery without toxicity to the mother or fetus (Young et al., 2022). Mitchell et al. showed that LNPs can target nucleic acid to fetuses without inducing fetal loss (Riley et al., 2021). They also designed ionizable LNPs for delivering Vascular Endothelial Growth Factor A mRNA to the placenta to induce vasodilation and treat placental insufficiency (Swingle et al., 2023). This study showed that LNP administration caused transient liver toxicity and placental inflammation, which resolved after 48 h. These findings suggest that LNPs may be a promising approach for treating placental dysfunction. Given the differences between SLNs and LNPs in chemical composition, administration routes, and biodistribution, it is crucial to assess the potential adverse effects of SLNs, particularly when repeatedly administered to women of reproductive age or pregnant individuals.

This study investigates the effects of repeated pre-mating administration of SLNs, unloaded or loaded with melatonin (SLN + mlt), a circadian rhythm regulator, lipophilic antioxidant, and potential anti-inflammatory agent (Reiter et al., 2016; Ferlazzo et al., 2020; Esposito and Cuzzocrea, 2010). The biocompatibility of SLN and SLN + mlt was assessed, focusing on potential toxicity to the maternal gastrointestinal tract, placenta, and fetus. We analyzed intestinal tight junction (TJ) markers and pro-inflammatory/anti-inflammatory cytokines, and evaluated pregnancy outcomes, including mating rate, embryo count, resorptions, embryo and placenta weights, and reproductive fitness of the first generation.

## 2 Materials and methods

### 2.1 Characterization of nanoparticle suspension

Solid Lipid Nanoparticles, with or without melatonin (SLN + mlt or SLN, respectively), were obtained from Nanovector S.r.l. (Turin, Italy). The complete SLN formulation consisted of water (citrate buffer, pH 5), glycerol, soy lecithin, glyceryl citrate/lactate/oleate/linoleate (E−472), glycerol monostearate (E−471), polysorbate 20, ascorbyl palmitate, sodium benzoate, α-tocopheryl acetate, strawberry flavor, and sucralose. The nanoparticle suspension contained 75 mg/mL of SLNs, with or without 1 mg/mL of melatonin (mlt), dispersed in citrate buffer (pH 5, Sigma-Aldrich, Darmstadt, Germany). The mean hydrodynamic diameter (dH) was 199.8 nm, with a polydispersity index (PDI) of 0.4 and a zeta potential of −41 mV.

### 2.2 Animal treatments

All animal procedures were approved by the Institutional Animal Care and Use Committee of the Tor Vergata University and the Italian Ministry of Health (approval prot. n. 262/2021-PR) and carried out according to the Italian and European rules (D.L.26/14; European Directive 2010/63/EU). 6 to 8 weeks old CD-1 female mice (Charles River Laboratories, Calco, Italy) were housed and mated under standard conditions with controlled light cycle (lights-on between 07:00 h and 19:00 h) and temperature (20°C). After 3 h fasting, 7.5 (low dose, LD) and 750 (high dose, HD) mg/kg of SLN + mlt were administered to groups of 18 mice each, via gavage in a final volume of 300 μL. As control, 8 mice were treated with 7.5 mg/kg of SLNs, 8 mice with 750 mg/kg of SLN and 18 mice received 300 μL of citrate buffer (CTRL). At the end of each exposure, animals were allocated back in their cages with water and food. The treatment was repeated three times a week for 6 weeks before mating. The day of the plug was considered day 0.5 of pregnancy (0.5 dpc). Half of the pregnant control and treated mice were sacrificed by cervical dislocation at 15.5 dpc. Uteri were collected and dissected using a stereomicroscope in order to carefully assess the presence of fetal resorptions or morphological alterations of both fetuses and placentas. Moreover, placentas and fetuses were weighted using an analytical balance and fetal crown-rump length was measured using a digital caliper. Maternal organs, including lung, liver, spleen, kidney, stomach and intestine, fetuses and placentas were collected for histological and real-time PCR analysis. The other half carried the pregnancies to term and the number of pups was recorded.

### 2.3 Hematology analysis

Blood samples were collected from half of the pregnant control and treated mice at 15.5 dpc, prior to sacrifice, via orbital sinus blood withdrawal. Approximately 100 μL of blood was obtained from each mouse. Complete blood counts were performed using an automated cell counter (Drew-3 Hematologic System, Drew Scientific, United States).

### 2.4 Tissue collection

At the time of sacrifice, placentas and fetuses were counted, measured, weighted using an analytical balance (Sartorius, Italy) and carefully observed under a stereomicroscope to screen for the presence of structural abnormalities. Maternal organs, fetuses and placentas were fixed in 4% paraformaldehyde solution for histological analysis or immediately frozen and stored at −80°C until use for protein and RNA extraction.

### 2.5 Reagents

All reagents were purchased from Sigma-Aldrich s. r.l. (Milano, Italy), unless otherwise specified.

### 2.6 Cell culture

The human colon adenocarcinoma cell line Caco-2 provided by American Type Culture Collection (ATCC) (Manassas, VA, United States) were expanded and maintained in Dulbecco’s Modified Eagle Medium (DMEM, Thermo Fisher Scientific; Waltham, MA, United States) supplemented with 20% fetal bovine serum (FBS), and 1% penicillin/streptomycin. For the viability assay, 24 h before the incubation with SLN with or w/o mlt, cells were seeded in cell media supplemented with 10% FBS at a density of 2.5 × 10^3^ cells/well in Falcon ^®^ 96-well Polystyrene Microplates (Corning Life Sciences, Chorges, France). For the viability and permeability assay on Caco-2 barrier model, 24 h before the treatment, cells were seeded on Transwell^®^ inserts (1 μm diameter pore-sizes, growth area of 4.2 cm^2^, Corning Life Sciences, Chorges, France) at a density of 38 ×10^4^ cells/insert in 10% FBS supplemented cell medium and cultured for 21 days to allow the differentiation of enterocyte-like cells. The culture medium was changed twice a week. All cell cultures were kept in a humidified incubator at 37°C in a 5% CO_2_ atmosphere.

### 2.7 Simulated human digestion system (SHDS)

*In vitro* digestion was performed according to the sequential digestion model described by Sohal et al., and slightly modified by Sohal et al. (2018), Marucco et al. (2020). The composition of the simulated digestive fluids (i.e., saliva, gastric, duodenal and bile fluids) is reported in Table 1. Briefly, simulated digestive fluids were added in sequential order at standard incubation times, as follows: 15 min for saliva, 4 h for gastric fluid and 4 h for intestinal fluid, composed of mixed duodenal and bile fluids. The ratio between fluids was 1:2:2:1 (saliva:gastric:duodenal:bile) with a final concentration of 0.5 mg/mL of SLNs. A full characterization of simulated human digestion fluid (SHDS)-treated SLNs has been previously reported (Antonello et al., 2022).

**TABLE 1:** Composition of simulated digestive fluids for the *in vitro* digestion model (amounts based on 100 mL of fluid).

Saliva fluid (pH 6.5 ± 0.1)	Gastric fluid (pH 1.4 ± 0.1)	Duodenal fluid (pH 8.1 ± 0.1)	Bile fluid (pH 8.0 ± 0.1)
Organic component (50 mL)			
20 mg Urea Milli-Q water	8.5 mg Urea 65 mg D-glucose 2 mg Glucuronic acid 33 mg D-Glucosamine hydrochloride Milli-Q water	25 mg Urea Milli-Q water	10 mg Urea Milli Q-water
Inorganic component (50 mL)			
89.6 mg KCl l 20 mg KSCN 102.2 mg NaH_2_PO_4_•H_2_O 57 mg Na_2_SO_4_ 0.18 mL 1 N NaOH 29.8 mg NaCl Milli-Q water	30.6 mg NH_4_Cl 40 mg CaCl_2_•2H_2_O 10.13 mg 1 N HCl 82.4 mg KCl 275.2 mg NaCl 30.6 mg NaH_2_PO_4_•H_2_O Milli-Q water	0.22 mL 1 N HCl 5 mg MgCl_2_•6H_2_O 56.4 mg KCl 8 mg KH_2_PO4 338.8 mg NaHCO_3_ 701.2 mg NaCl Milli-Q water	0.24 mL 1 N HC 37.6 mg KCl 578.5 mg NaHCO_3_ 525.9 mg NaCl Milli-Q water
Active component/enzymes			
5 mg Mucin 1.6 mg Uric acid 14.5 mg α-amylase	300 mg Mucin 100 mg BSA 100 mg Pepsin	300 mg Pancreatin 50 mg Lipase 100 mg BSA 22.2 mg CaCl_2_•2H2O	600 mg Bile 180 mg BSA 20 mg CaCl_2_•2H2O

KCl, Potassium chloride; KSCN, Potassium thiocyanate; NaH_2_PO_4_•H_*2*_O, Sodium phosphate monobasic monohydrate; Na_2_SO_4_, Sodium sulfate; NaCl, Sodium chloride; NaOH, sodium hydroxide; NH_4_Cl, ammonium chloride; CaCl_2_•2H_2_O, Calcium chloride dihydrate; HCl, hydrochloric acid; MgCl_2_•6H_2_O, Magnesium chloride hexahydrate; KH_2_PO_4_, Potassium phosphate monobasic; NaHCO_3_, Sodium bicarbonate; BSA, Bovine serum albumin.

### 2.8 Cell viability

Caco-2 cells were exposed for 24 h to pristine and SHDS-treated SLNs the day after cell seeding. The desired concentrations of SLNs (2.5, 5, 10, 20, 50, 100 and 150 μg/mL) were obtained by diluting the stock suspensions in cell culture medium. Cell viability was evaluated using the 2-(4-iodophenyl)-3-(4-nitrophenyl)-5-(2,4-disulfophenyl)-2H-tetrazolium, monosodium salt (WST-1) assay. Caco-2 cells cultured in 96-well plates were incubated with the reagent for 2 h, while Caco-2 cells cultured on the transwells were incubated for 30 min, according to the manufacturer’s instructions. Trans Epithelial Electrical Resistance (TEER).

Caco-2 cells were grown on Transwell inserts for 21 days. Intestinal barrier formation was assessed by measuring the TEER with Millicel ERS-2 Voltohmmeter (Merck KGaA, Darmstad, Germany). Only monolayers with TEER values >600 Ω*cm^2^ were used for subsequent experiments. The resistivity was calculated subtracting the values obtained from a cell-free insert to values obtained from inserts with cells and multiplying it by the growth area. TEER measurements were also recorded after incubating the Caco-2 barrier with either pristine or SHDS-treated SLNs for 24 h at the concentration of 50 μg/mL.

### 2.9 Barrier permeability

Barrier integrity was assessed using the Lucifer Yellow (LY) test following the Nanoreg Standard Operating Procedure. Briefly, the basolateral (Bl) and apical (Ap) medium of each well were removed, and the wells were washed three times with pre-warmed (37°C) Hanks’ Balanced Salt Solution (HBSS, Thermo Fisher Scientific; Waltham, MA, United States). Inserts were then transferred into new 6-wells plates containing 1.5 mL of HBSS; 0.5 mL of HBSS containing 0.4 mg/mL of LY were added in the apical compartment and incubated for 2 h at 37°C in 5% CO^2^ incubator. The Bl medium was then collected and loaded in duplicate into black 96-well plates and the fluorescence was evaluated using a Synergy HTX microplate reader (Bio-Tek Instruments) (excitation wavelength 428 nm, emission 536 nm). Apparent barrier permeability (Papp) expressed as cm/s was calculated as follows:

$$\text{Papp}=\left(\left(\Delta Q/\Delta t\right)\cdot V\right)\cdot \left(1/\text{AC}0\right)$$

where ΔQ/Δt ((mg/mL)/s) is the SLNs transport rate from the Ap to the Bl chamber, A (cm^2^) is the area of the membrane (which in our case was 4.2 cm^2^), and C0 (mg/mL) is the initial SLNs concentration in the Ap chamber.

### 2.10 Quantitative real time PCR (qRT-PCR)

RNA from cell cultures was prepared using the Ribozol Reagent (VWR; Radnor, PA) according to the manufacturer’s protocol. mRNA was reverse transcribed using the iScript cDNA synthesis kit (Bio-Rad, Segrate, Italy), following the manufacturer’s specifications. Gene expression was measured using Bio-Rad Laboratories’ iTaq Universal SYBR Green Supermix. qRT–PCR was performed using a CFX96 Real-Time System (Bio-Rad Laboratories). Differences of gene expression were quantified using the ΔΔCt method with normalization to *S14* (for human genes) and *actb* (for murine genes). Specific murine and human primer sequences are listed in Table 2, Table 3, respectively.

**TABLE 2:** Human primer sequences.

Gene name	Strand	Sequences
*TNFα*	FW	5′- TGG​GAT​CAT​TGC​CCT​GTG​AG
	RV	5′- GGT​GTC​TGA​AGG​AGG​GGG​TA
*IL-6*	FW	5′- GGT​ACA​TCC​TCG​ACG​GCA​TCT
	RV	5′- GTG​CCT​CTT​TGC​TGC​TTT​CAC
*IL-10*	FW	5′- AGA​CAG​ACT​TGC​AAA​AGA​AGG​C
	RV	5′- TCG​AAG​CAT​GTT​AGG​CAG​GTT
*IL-22*	FW	5′- GCTGCCTCCTTCTCTTGG
	RV	5′- GTG​CGG​TTG​GTG​ATA​TAG​G
^a^ *S14*	FW	5′- AGG​TGC​AAG​GAG​CTG​GGT​T
	RV	5′- TCC​AGG​GGT​CTT​GGT​CCT​ATT​T

^a^ Housekeeping gene.

**TABLE 3:** Murine primer sequences.

Gene name	Strand	Sequences
*Tnfα*	FW	5′- CCT​CTC​ATG​CAC​CAC​CAT​CA
	RV	5′- GCA​TTG​CAC​CTC​AGG​GAA​GA
*Il-1β*	FW	5′- AAG​GGG​ACA​TTA​GGC​AGC​AC
	RV	5′- ATG​AAA​GAC​CTC​AGT​GCG​GG
*Il-6*	FW	5′- AGC​CCA​CCA​AGA​ACG​ATA​GTC
	RV	5′- GCA​TCA​GTC​CCA​AGA​AGG​CA
*Il-10*	FW	5′- ACC​TGG​TAG​AAG​TGA​TGC​CC
	RV	5′- ACA​CCT​TGG​TCT​TGG​AGC​TT
*Il-22*	FW	5′- GAC​AGG​TTC​CAG​CCC​TAC​AT
	RV	5′- TCG​CCT​TGA​TCT​CTC​CAC​TC
*Nos2*	FW	5′- AGG​TTG​TCT​GCA​TGG​ACC​AG
	RV	5′- GCT​GGG​ACA​GTC​TCC​ATT​CC
*Cldn2*	FW	5′- AGG​AAT​TGC​CCA​GAA​GCC​AA
	RV	5′- GGT​TTA​GCA​GGA​AGC​TGG​GT
*Cldn3*	FW	5′- GGT​GAC​AGA​CGA​CAC​ACA​GT
	RV	5′- GTC​CAT​TCG​GCT​TGG​ACA​GT
*Cldn4*	FW	5′- TGG​TGT​GCT​GAG​TGA​CTG​AC
	RV	5′- GGG​TCA​AGC​ACA​GTC​ATT​GC
*Cldn5*	FW	5′- TTA​AGG​CAC​GGG​TAG​CAC​TC
	RV	5′- CAA​CGA​TGT​TGG​CGA​ACC​AG
*Ocln*	FW	5′- TCT​TTC​CTT​AGG​CGA​CAG​CG
	RV	5′- AGA​TAA​GCG​AAC​CTG​CCG​AG
*Tjp1*	FW	5′- AGA​GCT​ACG​CCT​GGA​GAT​TC
	RV	5′- TGT​CCT​ATT​TCC​AGC​TCC​CG
*Muc3*	FW	5′- GCA​GAA​GGG​CGA​TAA​GTG​GT
	RV	5′- GCT​GAC​ATT​TGC​CGT​AGC​TG
^a^ *Actb*	FW	5′-TGA​AGT​GTG​ACG​TTG​ACA
	RV	5′-TAG​AAG​CAC​TTG​CGG​TGC​ACG

^a^ Housekeeping gene.

### 2.11 Statistical analysis

Statistical analysis was performed using the GraphPad Prism (version 7.00) software. Data were analysed using either parametric (ANOVA test) or nonparametric (Kruskal–Wallis test) tests, based on the results of variance analysis (Brown-Forsythe or F test). Data are presented as mean ± standard error mean (SEM). Asterisks indicate the level of statistical significance: *p < 0.05; **p < 0.01; ***p < 0.001; ****p < 0.0001. For multiple groups comparison, different letters are used to denote statistically significant differences, while the same letter indicate that differences are not statistically significant.

## 3 Results

### 3.1 Effects of maternal exposure to SLN with or w/o melatonin

SLN + mlt and SLN were administered via gavage to CD-1 female mice at the concentrations of 7.5 (low dose, ^LD^SLN + mlt or ^LD^SLN) or 750 (high dose, ^HD^SLN + mlt or ^HD^SLN) mg/kg, three times a week for 6 weeks before mating. Mating rate was recorded during a period of 7 days and no significant differences in the number of plugged females were observed between CTRL and SLN- or SLN + mlt-treated mice (Table 4).

**TABLE 4:** Mating rated recorded during a period of 7 days for CTRL and loaded and unloaded SLN-treated mice.

Day	CTRL (n = 18)	^LD^SLN (n = 8)	^LD^SLN + mlt (n = 18)	^HD^SLN (n = 8)	^HD^SLN + mlt (n = 18)
1	4 (22.22%)	2 (25%)	5 (27.78%)	2 (50%)	3 (16.67%)
2	2 (11.11%)	1 (12.5%)	3 (16.67%)	2 (50%)	2 (11.11%)
3	3 (16.67%)	1 (12.5%)	0	0	3 (16.67%)
4	4 (22.22%)	1 (12.5%)	4 (22.22%)	0	6 (33.33%)
5	4 (22.22%)	1 (12.5%)	6 (33.33%)	1 (12.5%)	3 (16.67%)
6	0	2 (25%)	0	2 (25%)	1 (5.55%)
7	1 (5.55%)	0	0	1 (12.5%)	0

**FIGURE 1:**
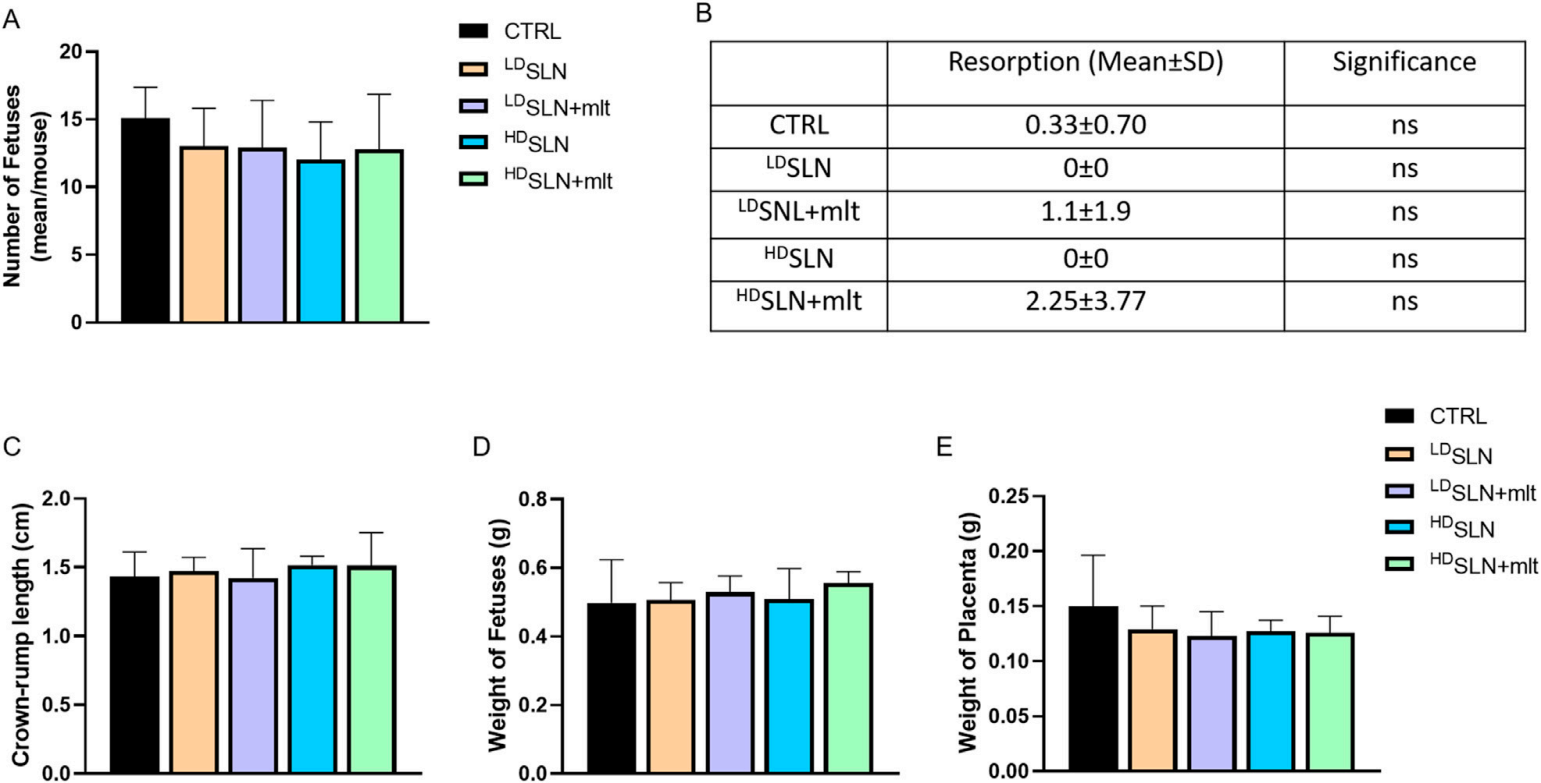
Effect of loaded and unloaded SLNs on pregnancy outcome. Number of fetuses **(A)** and fetal resorptions **(B)** from controls and melatonin loaded and unloaded SLN treated mothers. Fetal crown-rump length **(C)** and weight of fetuses **(D)** and placentas **(E)** from control and SLN-treated mothers. Statistical analysis was performed using ANOVA **(A, B)** and Kruskal–Wallis **(C–E)** tests.

At 15.5 dpc, half of the females from each group were sacrificed, and fetuses and placentas were analysed. The number of fetuses was comparable across all groups (Figure 1A), with a slight reduction in the ^HD^SLN-treated group compared to controls; however, statistical significance was not met (*p* > 0.05). Although few fetal resorptions were observed in CTRL and SLN + mlt treated mice, but not in the SLN treated groups, the difference did not reach statistical significance (Figures 1B, C).

**FIGURE 2:**
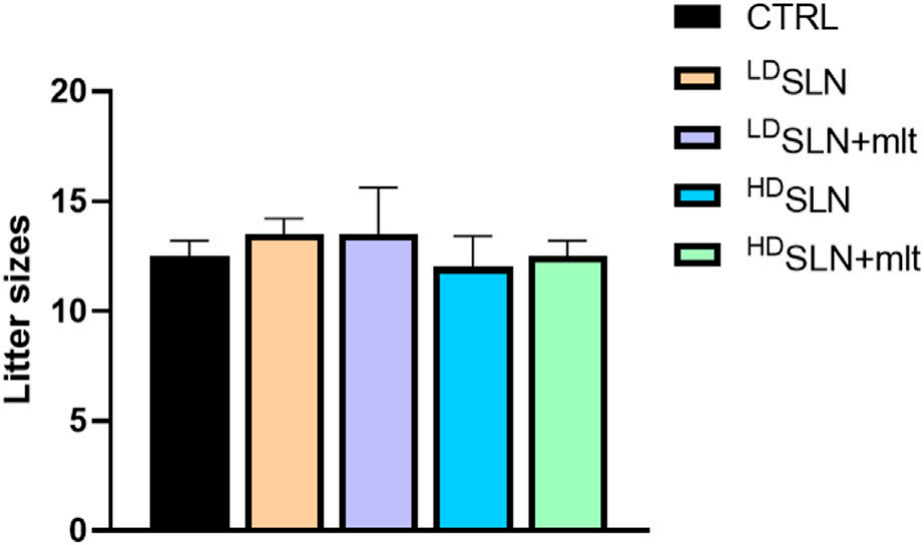
Number of newborns per litter in the control and SLN-treated mothers. Statistical analysis was performed using Kruskal–Wallis test.

No morphological alterations were observed in placentas and fetuses, nor was a statistically significant difference detected in crown-rump length or fetal and placental weight following maternal exposure to any of the studied particles, as shown in Figures 1C–E. Half of the dams was left to give birth, and litter size evaluated. No differences in the number of newborns per litter were observed among the five groups (Figure 2; *p* > 0.05).

**FIGURE 3:**
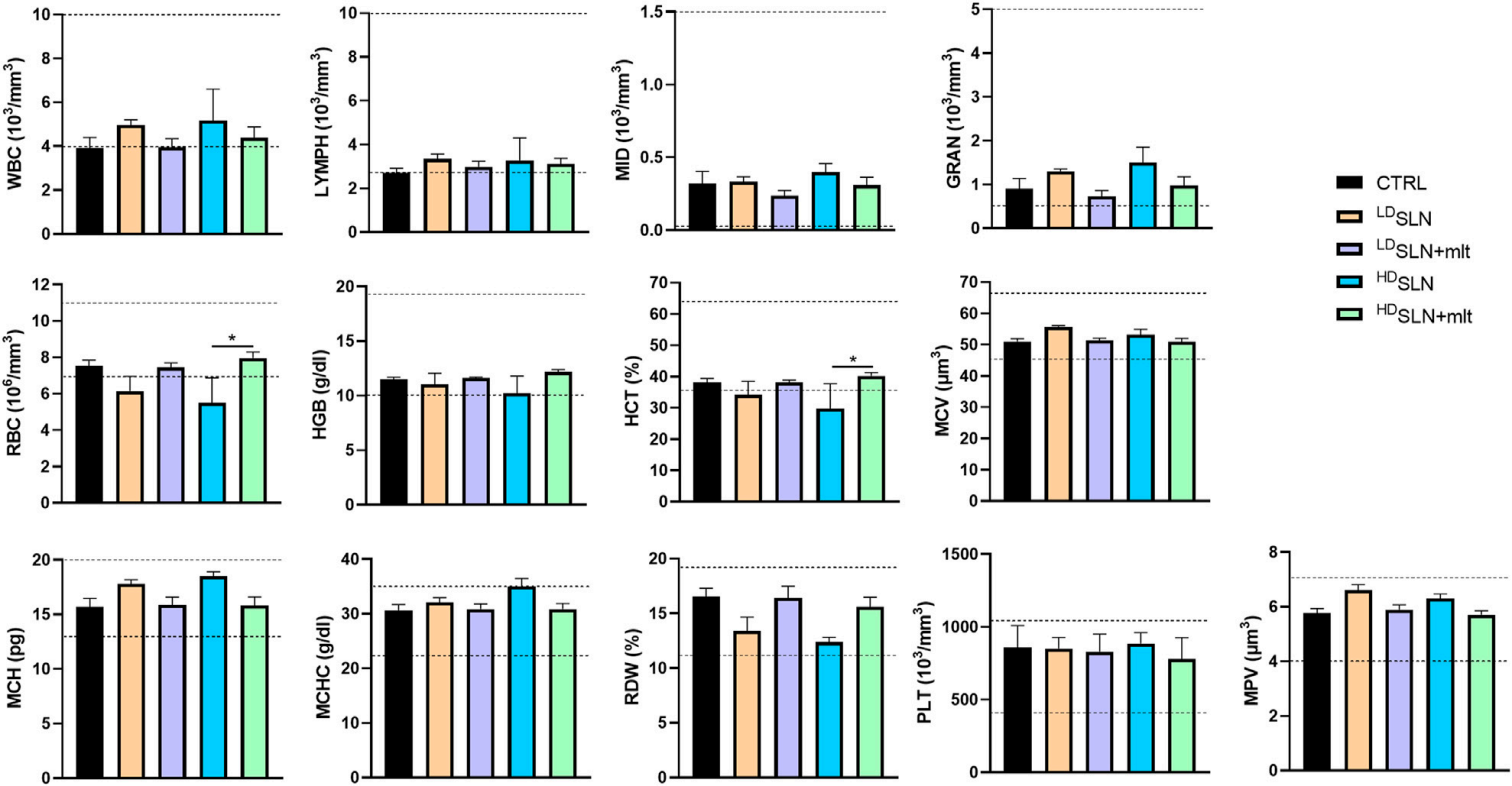
Blood cell counts of 15.5 dpc pregnant female treated with the different types of particles at the different concentrations tested. Dotted lines indicate reference ranges. Statistical analysis was performed using ANOVA test. Abbreviations: WBC, White Blood Cells, LYMPH, Lymphocytes; MID, Middle-sized cells; GRAN, Granulocytes; RBC, Red Blood Cells; HGB, Haemoglobin; HCT, Haematocrit; MCV, Mean Cell Volume; MCH, Mean Corpuscular Haemoglobin; MCHC, Mean Corpuscular Haemoglobin Concentration; RDW, Red blood cell Distribution Width; PLT, Platelets; MPV, Mean Platelet Volume.

In the group of pregnant females sacrificed at 15.5 dpc, the effect of oral administration of SLN or SLN + mlt on haematological parameters was also evaluated. As shown in Figure 3, no significant changes in blood cell counts were observed among the groups, except for red blood cells (RBC) and haematocrit (HCT), which were reduced in ^HD^SLN group compared to ^HD^SLN + mlt group.

### 3.2 Effect of melatonin loaded and unloaded SLNs on different tracts of maternal small intestine

**FIGURE 4:**
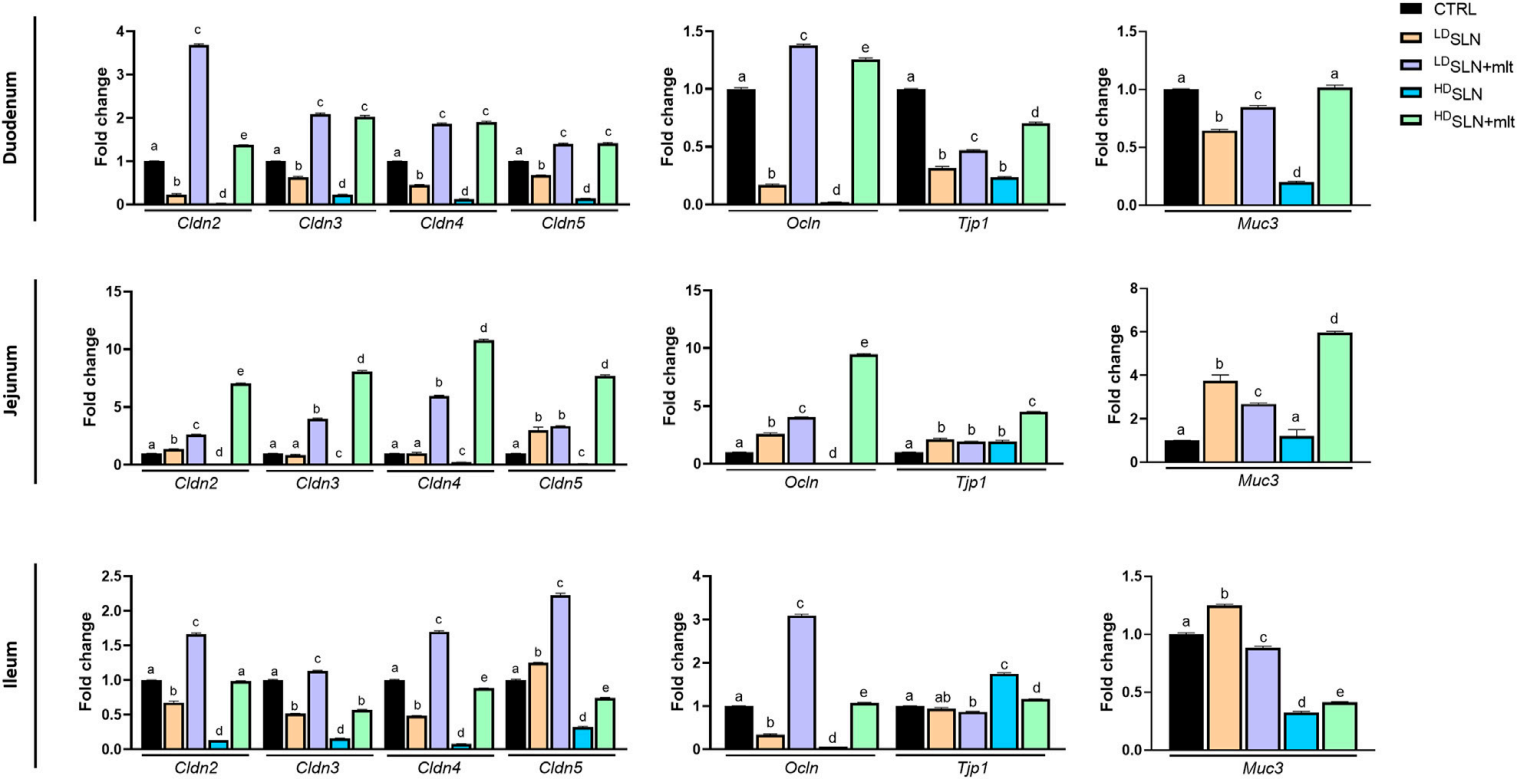
Evaluation of the intestinal barrier in control, loaded and unloaded SLN-treated mothers. qRT-PCR analysis for Claudins (*Cldn*) 2, 3, 4, and 5, Occludin (*Ocln*), Tight junction protein-1 (*Tjp1*) and Mucin 3 (*Muc3*) in duodenum, jejunum and ileum obtained after sacrifice from control and treated pregnant mice at 15.5 dpc. Statistical analysis was performed using one-way ANOVA test. Different letters indicate statistically significant differences.

The effects of the different types and concentrations of SLNs on the maternal small intestine were investigated in terms of barrier integrity and inflammation. qRT-PCR analysis of genes encoding TJ proteins, mucin, and pro- and anti-inflammatory cytokines was performed on the three different sections of the small intestine, i.e., duodenum, jejunum, and ileum, collected from the pregnant females sacrificed at 15.5 dpc. In the duodenum, the expression of all studied TJ genes, except for *Tjp1*, showed a similar trend (Figure 4). Indeed, treatment with SLNs induced a dose-dependent downregulation of all claudins and of *Ocln*, while SLN + mlt treatment induced a significant upregulation of the same genes (Figure 4, upper panel). In contrast, *Tjp1* was downregulated in all conditions, although to a lesser extent in the SLN + mlt treated groups. Similarly, the expression of the membrane-associated intestinal mucin, *Muc3*, was significantly downregulated by SLN treatment in a dose-dependent manner, as well as by ^LD^SLN + mlt treatment. However, in the ^HD^SLN + mlt group, *Muc3* expression was comparable to controls (Figure 4). With respect to the jejunum, the expression of all studied genes was significantly upregulated in the SLN + mlt treated groups in a dose-dependent manner. In contrast, ^HD^SLNs induced a significant downregulation of all claudins and of *Ocln*, while *Tjp1* was upregulated and *Muc3* expression was unaffected. At low dose, the unloaded nanoparticles upregulated the expression of all genes, except for *Cldn3* and *Cldn4*, which were comparable to the controls (Figure 4, middle panel).

In the ileum, treatment with ^LD^SLN + mlt resulted in the increased expression of all claudins and of *Ocln*, while *Tjp1* and *Muc3* expression were reduced (Figure 4, lower panel). Unlike the previous gut tracts, ^HD^SLN + mlt treatment induced slight variations of all genes (up or downregulations), with the exception of *Cldn3* and *Muc3*, whose expression was reduced by half (Figure 4, lower panel). ^LD^SLNs significantly downregulated *Cldn2*, *Cldn3*, *Cldn4* and *Ocln*, but induced a slight increase in *Cldn5* and *Muc3* expression, and no change in *Tjp1*. ^HD^SLNs significantly downregulated all genes except *Tjp1*.

The expression of pro-inflammatory (i.e., *Il-1β, Il-6, Tnfα* and *Nos2*) and anti-inflammatory (i.e., *Il-22* and *Il-10*) cytokines in the same samples was also investigated.

**FIGURE 5:**
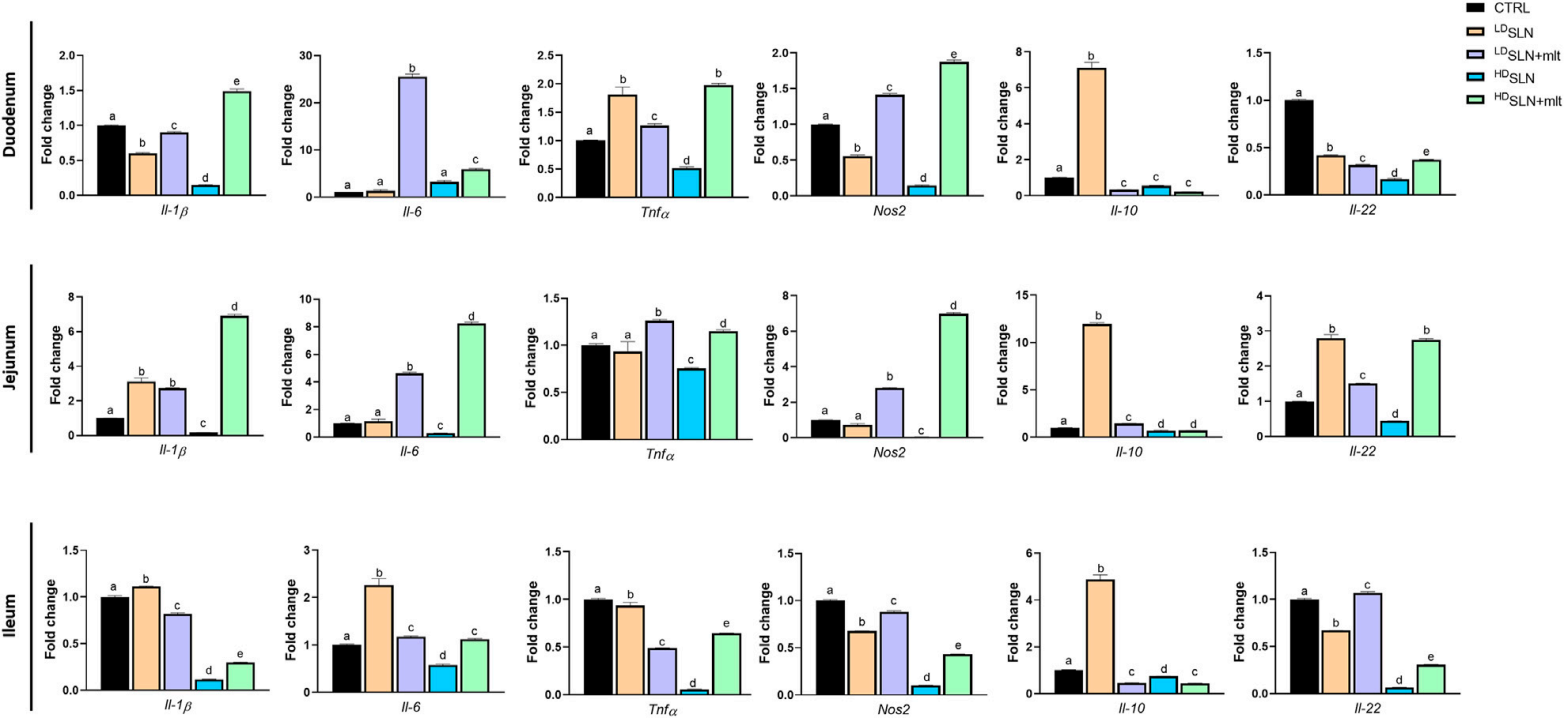
Evaluation of inflammation related genes in different intestinal tracts taken from control, SLN- and SLN + mlt-treated mothers. qRT-PCR analysis for *Interleukin (Il) 1β, 22, 6,* and *10*, *Tumor necrosis factor alpha (Tnfα)*, and *Nitric oxide synthase 2 (Nos2)* in duodenum, jejunum and ileum obtained from dams sacrificed at 15.5 dpc. Statistical analysis was performed using one-way ANOVA test. Different letters indicate statistically significant differences.

In the duodenum, the expression of the pro-inflammatory cytokines *Il-1β* and *Nos2* was significantly downregulated by SLN treatment s, independently of the dose, while *Il-6* expression remained unchanged. *Tnfα* was significantly upregulated at the low dose but downregulated at high dose. The anti-inflammatory cytokine *Il-22* was significantly downregulated at both doses of SLNs, while *Il-10* was downregulated at the high dose, but significantly upregulated at the low dose. In the SLN + mlt, all pro-inflammatory cytokines were significantly upregulated at both doses, except for *Il-1β*, which was slightly reduced at the low dose. Conversly, SLN + mlt significantly downregulated all the anti-inflammatory cytokines independently of the dose (Figure 5, upper panel). In the jejunum, ^LD^SLNs treatment had no effect on the pro-inflammatory cytokines, except for Il-1β, whose expression was significantly upregulated. At the high dose, all cytokines were significantly downregulated. The anti-inflammatory cytokines were significantly upregulated at low dose and downregulated at high dose. In the SLN + mlt-treated groups, the expression of all pro-inflammatory cytokines was upregulated, dose-dependently,except for *Tnfα,*. Concerning the anti-inflammatory cytokines, both doses of SLN + mlt upregulated *Il-22* in a dose dependent manner, while *Il-10* was slightly upregulated in the ^LD^SLN + mlt treated group and slightly downregulated at the high dose (Figure 5, middle panel). Regarding the expression of cytokines in the ileum, following maternal administration of SLN and SLN + mlt, all pro-inflammatory cytokines were significantly downregulated, with the exception of a slight up-regulation of *Il-1β* and a two-fold increase of *Il-6* in the ^LD^SLNs group and a very slight increase of *Il-6* in the SLN + mlt group at both doses. Similarly, the two studied anti-inflammatory cytokines were downregulated except for a significant increase in *Il-10* expression in the ^LD^SLN group and very slight increase of *Il-22* in the ^LD^SLN + mlt group (Figure 5, lower panel).

### 3.3 *In vitro* cytotoxicity assessment

**FIGURE 6:**
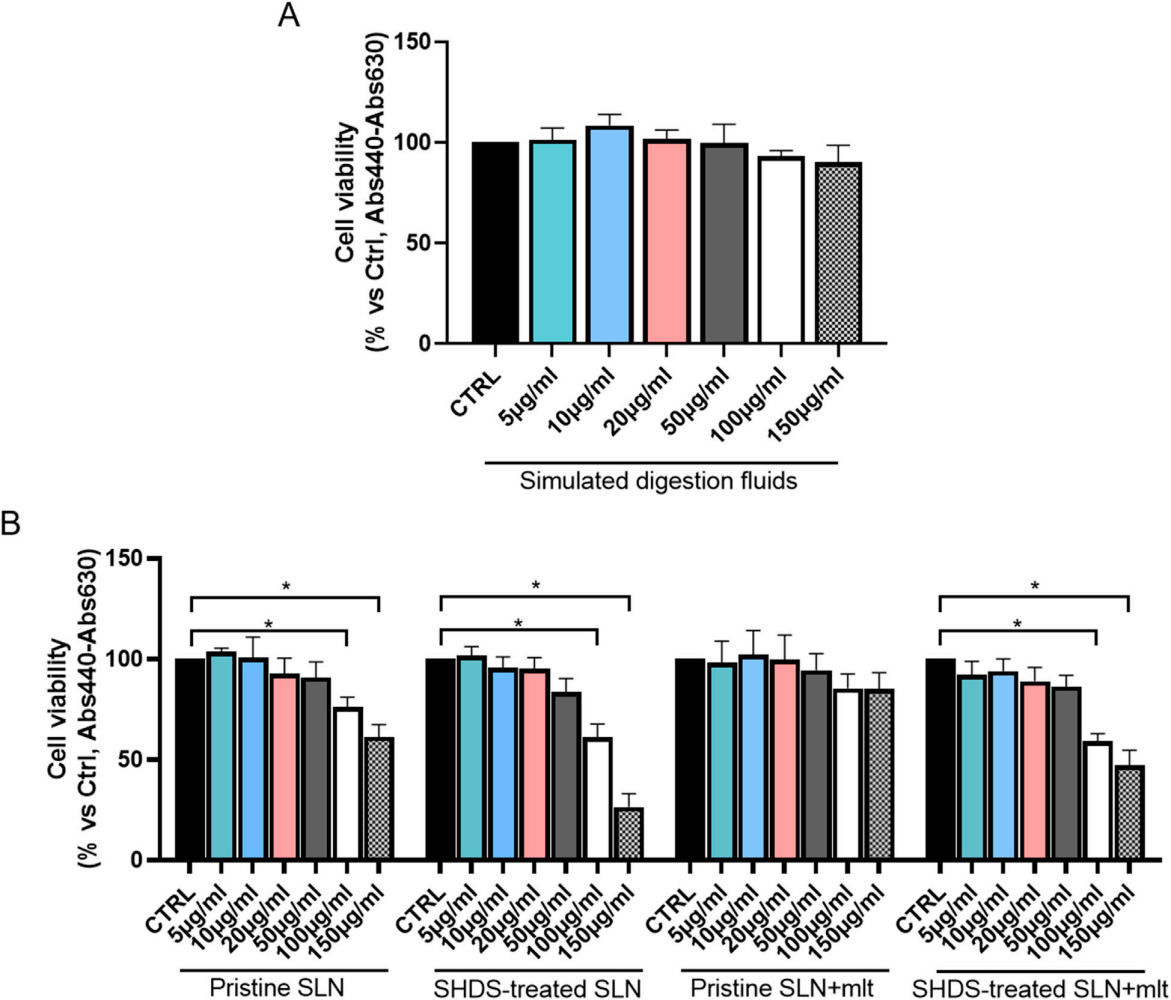
Evaluation of cell viability upon exposure to increasing concentrations of **(A)** simulated digestion fluids and **(B)** melatonin loaded and unloaded pristine and SHDS-treated SLNs. Statistical analysis was performed using one-way ANOVA test.

**FIGURE 7:**
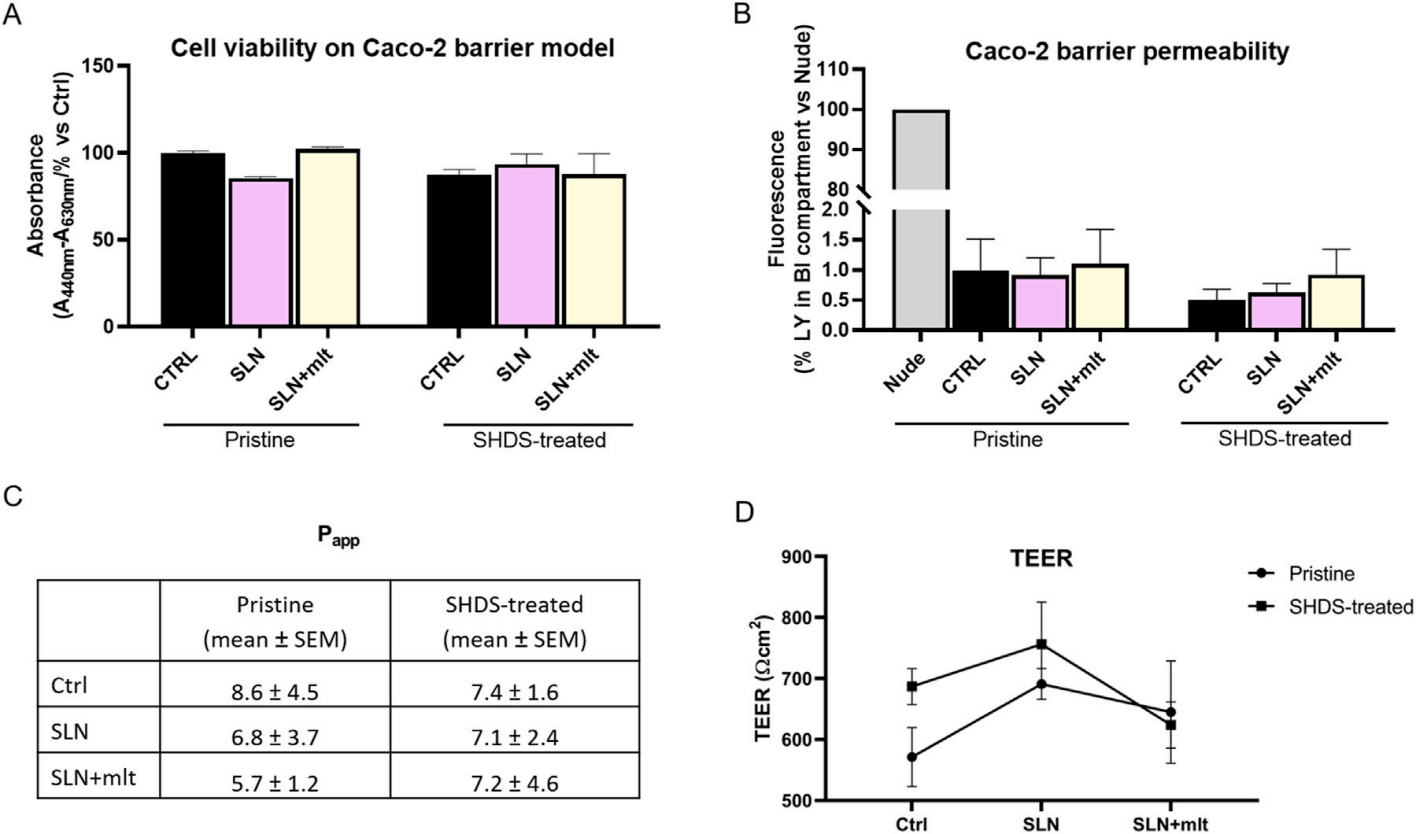
Assessment of Caco-2 cell barrier integrity following exposure to the particles under study. **(A)** cell viability, **(B)** barrier permeability, **(C)** apparent permeability (Papp) expressed as cm/s x 10^8^ and **(D)** TEER measurements were evaluated on Caco-2 cells grown for 21 days on porous membrane inserts and then incubated for 24 h with 50 μg/mL pristine and SHDS-treated melatonin loaded and unloaded particles. Statistical analysis was performed using one-way ANOVA test.

**FIGURE 8:**
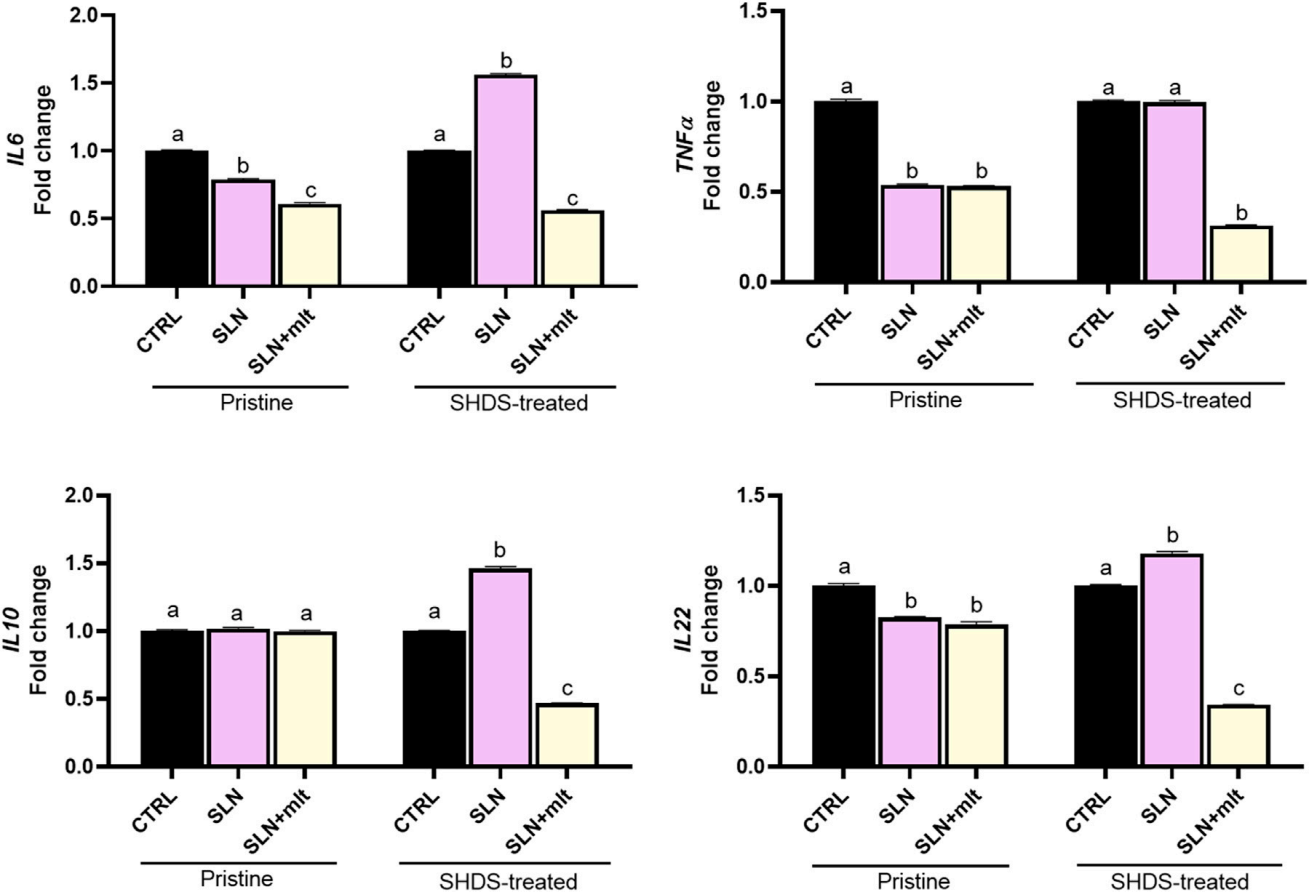
Expression of pro-inflammatory and anti-inflammatory cytokines following exposure of the Caco-2 barrier to the pristine and SHDS-treated melatonin loaded and unloaded particles. Statistical analysis was performed using one-way ANOVA test. Different letters indicate statistically significant differences.

As previously reported, the digestive process may affect the bio-identity of nanoparticles, altering their physico-chemical properties and hence their effects on gut cells (Antonello et al., 2022). For this reason, we treated SLN and SLN + mlt with the SHDS and assessed their biocompatibility *in vitro* (Soliman et al., 2024) on undifferentiated intestinal Caco-2 cells. These cells were exposed to concentrations ranging from 5 to 150 μg/mL, a range in which the simulated digestive fluids had no effect on cell viability (Figure 6A). Pristine SLN + mlt did not exhibit any toxicity across the tested concentration range, while SHDS-treated SLN + mlt showed a significant reduction in cell viability at the concentrations of 100 and 150 μg/mL (Figure 6B). As observed *in vivo*, SLNs were slightly more toxic than the mlt-loaded nanoparticles, as cell viability significantly decreased at 100 μg/mL for both pristine and SHDS-treated SLNs (Figure 6A). This higher cytotoxicity is likely due to the absence of mlt that is recognized as a factor maintaining the homeostasis of intestinal epithelium (Peters et al., 2024; Gao et al., 2019). The highest non-toxic concentration of SHDS-treated SLN + mlt, i.e., 50 μg/mL, was used to investigate the effects of pristine and SHDS-treated SLN + mlt on the integrity of the intestinal barrier model. This model was established by culturing Caco-2 cells for 21 days on porous membrane inserts to allow their differentiation into enterocyte-like cells (Peterson and Mooseker, 1992). After exposure to pristine or the SHDS-treated SLNs, cell viability of the simulated gut barrier was assessed by the WST-1 assay. No effects were recorded for both type of nanoparticles (Figure 7A). Barrier integrity was further assessed by measuring the translocation of LY, and TEER. Neither the LY assay, evaluated as both percentage of translocated LY (Figure 7B) and Papp (Figure 7C), nor the TEER measurements (Figure 7D) showed significant differences in any condition tested. To further investigate the effect of the SLNs on the simulated gut barrier, the expression of two pro-inflammatory cytokines (i.e., *TNFα* and *IL6*) and two anti-inflammatory cytokines (i.e., *IL10* and *IL22*) was investigated. The expression of both *IL6* and *TNFα* was reduced when cells were exposed to pristine loaded and unloaded particles. For the SHDS-treated particles, this reduction was observed only for the mlt loaded particles, while the unloaded particles slightly upregulated *IL6* and did not affect *TNFα* (Figure 8). Concerning the expression of the anti-inflammatory cytokines, pristine particles had no or very mild effects, while both *IL10* and *IL22* show a similar trend after barrier exposure to the SHDS-treated particles. SLN treatment resulted in a slight upregulation of *IL10* and *IL22*, while SLN + mlt induced a more pronounced decrease in these cytokines (Figure 8).

## 4 Discussion

This study aimed to comprehensively investigate the potential effect of lipid-based nanocarriers, specifically SLNs, on reproductive outcomes. We focused on determining whether the repeated oral administration of SLNs to female mice prior to mating would influence key pregnancy outcomes. The rationale behind this experimental design was to assess whether exposure of women in reproductive age to drug-delivering nanocarriers could potentially affect their reproductive health. Indeed, while women are generally cautious about drug use during pregnancy, their pre-pregnancy behaviors may still reflect in later detrimental effects.

Very few data about the effect of premating exposure to SLNs on pregnancy outcome are present in the literature. Bowman et al., in 2021 reported on the intramuscular administration of lipid nanoparticles in female rats twice prior to mating to vehicle the mRNA encoding the severe acute respiratory syndrome coronavirus 2 (SARS-CoV-2) spike protein (the commercially available BNT162b2). They reported no effects of this formulation on female mating performance, fertility, or any ovarian or uterine parameters, nor on embryo-fetal or postnatal survival, growth, physical development or neurofunctional development in the offspring through the end of lactation; however, in this study information on NP physicochemical properties were not provided and administration of the particles was performed only 21 and 14 days prior to mating (Bowman et al., 2021). In the present study, to explore the effect of repeated premating administration of SLNs on pregnancy, we orally administered SLNs at two different dosages—low (7.5 mg/kg) and high (750 mg/kg) —three times a week for 6 weeks to CD1 female mice. These females were subsequently mated with males of proven fertility, which were not exposed to the SLNs, ensuring that any observed effects were solely due to the maternal exposure to the nanoparticles. Pregnancy was monitored from conception to delivery, and a range of pregnancy parameters were assessed. All parameters, including time to pregnancy, pregnancy duration, litter size, and the presence of any gross anomalies in the pups, were not significantly affected by SLN administration.

In addition to these, we evaluated the potential effects of SLNs on embryonic development. This included examining the number of implantation sites, fetus count, incidence of fetal resorptions, and measurements of crown-rump length, as well as fetal and placental weights. For these parameters, no significant effects of SLN administration were observed. To assess whether repeated SLN administration could affect maternal health, we analysed key biochemical parameters to identify any potential systemic impacts of SLN on maternal physiology. Although statistically significant differences were observed between the groups, all values remained within normal physiological ranges, suggesting that the repeated premating oral administration of SLNs had no overt adverse impact on maternal health. Our findings align with previous studies that reported no adverse effects of lipid-based nanoparticles, including those loaded with nucleic acids, on both maternal and fetal health (Young et al., 2022; Riley et al., 2021; Swingle et al., 2023). Very recently, the importance of developing safe-by-design lipid based nanoparticles to deliver mRNA to both the placenta and nonreproductive maternal organs has been highlighted, with no fetal delivery and no effects on pup development (Chaudhary et al., 2024).

Given that oral administration is a common route for drug delivery, we also assessed the impact of SLNs on the GIT and its epithelial barrier integrity. Gross histological examination of the GIT collected from treated mothers revealed no adverse effects on the epithelial barrier or underlying tissues. The lack of adverse effects on the mother and on pregnancy parameters suggests either of a high biocompatibility of the particles or their low GIT absorption. To further asses this, we studied the expression of molecules regulating epithelial permeability in the duodenum, jejunum and ileum of dams at 15.5 dpc. These molecules included the cell-to-cell junction proteins claudins, occludin and the tight junction protein 1 (*Tjp1* or *zonula occludens1, Z O -1*), as well as the intestinal mucosa associated mucin glycoprotein *Muc3*. In all three gut sections, we observed that the majority of the studied genes were significantly downregulated by the unloaded SLNs, generally in a dose-dependent manner. However, the presence of mlt not only counteracted such decrease, but often resulted in a significant upregulation, likely due to its multiple beneficial effects (Srinivasan et al., 2011; Majidinia et al., 2017; Hill et al., 2015; Reiter et al., 2010). Interestingly, although the unloaded particles may affect gut permeability, this was not reflecting in adverse effects on pregnancy. This result suggests that administering these nanoparticles as a drug delivery vehicle, at the doses we used—equivalent to the amount of mlt a human might consume—would not pose a risk to future pregnancies, although it might compromise the integrity of the intestinal epithelium. In this respect, considering that oral administration of drugs could be associated to inflammation of the GIT, we assessed the expression of genes encoding for pro- and anti-inflammatory molecules in the same biopsies from treated mothers. The majority of the studied pro-inflammatory cytokines were affected by treatment with both unloaded and loaded SLNs at the two doses used, with a modulation of 1.5 to 2 folds. Interestingly, with the exception of *Il-6*, which appeared strongly upregulated in the duodenum of mothers treated with ^LD^SLN + mlt, the highest increase in the expression of pro-inflammatory cytokines was observed in the jejunum. Indeed, in the jejunum the expression of *Il-1β, Il-6* and *Nos2* was significantly upregulated by both mlt loaded and unloaded particles. Although no literature data on intestinal inflammation associated to oral administration of SLNs in pregnancy are available, it is well documented that oral administration of other types of nanoparticles could be associated to the development of colitis and intestinal inflammation marked by significant histological transformations (Ogawa et al., 2021; Sousa et al., 2023), which however were not observed in our samples. NOS2 may be considered an inflammation-promoting factor because its proper activation in the inner lining of the bowels and their blood vessels leads to the production of nitric oxide (NO). NO is crucial for the optimal functioning of the digestive tract as it promotes fluid secretion, enhances blood flow, maintains barrier integrity, and eliminates infectious bacteria and cancer cells. The production of NOS2 is regulated by T cell-derived cytokines: while TNF-α, IL-1β, and IFN-γ activate NOS2, the anti-inflammatory cytokines IL-4 and IL-13 inhibit its activity. If NOS2 is not properly regulated by these factors, its excessive activity can contribute to the onset and progression of conditions such as inflammatory bowel disease (IBD) (Boughton-Smith et al., 1993; Singer et al., 1996; Rachmilewitz et al., 1998). The anti-inflammatory cytokines studied, namely, *Il-10* and *Il-22*, exhibited different responses. *Il-10* expression was only marginally reduced by the treatments, except for ^LD^SLN, which led to a significant increase in its expression across all three intestinal segments. *Il-22* gene expression was also minimally affected by the treatments, with the most pronounced modulation occurring in the jejunum, where a more than two-fold increase was observed following treatment with ^LD^SLN and SLN + mlt at both tested doses. This increase in *Il-22* may counterbalance the elevated levels of pro-inflammatory cytokines, potentially explaining the absence of significant histological alterations and the maintenance of the barrier integrity. No gross histological alterations were observed also in the liver (not shown). To further substantiate these findings, we performed *in vitro* studies using a well-established model of the intestinal epithelial barrier (Antonello et al., 2022). This model allowed to compare the effects of pristine (unloaded and loaded) SLNs with particles treated with the SHDS (Sohal et al., 2018; Marucco et al., 2020), that reproduce *in vitro* the process of digestion and that it is well-known to drive modifications of nanoparticles (Fadeel et al., 2013; Hayder et al., 2020; Shi et al., 2020; Zhou et al., 2021). The results indicated that even after the SHDS treatment, at reasonable concentrations, both loaded and unloaded SLNs did not affect the integrity of the gut epithelial barrier, further confirming their biocompatibility. These data further corroborate the absence of major effects on gut barrier integrity observed *in vivo*, despite the observed increase in pro-inflammatory mediators. *In vitro*, after the exposure of the Caco-2 barrier to pristine and SHDS-treated melatonin loaded and unloaded particles, the expression of the pro-inflammatory cytokines *IL6* and *TNFα* was unchanged or reduced, with the exception of SHDS-treated SLNs which induced a mild increase in the expression of *IL6*. The expression of the anti-inflammatory cytokines was also slightly affected, with both *IL10* and *IL22* showing a similar trend for the SHDS-treated particles. These data indicated low effects of the studied particles on intestinal inflammatory status and further confirmed the absence of effects on intestinal barrier integrity. However, it should be mentioned that, apart from melatonin, delivering other drugs that may induce oxidative stress, such as doxorubicin and others anti-cancer drugs (Norouzi et al., 2020; Sharma et al., 2007), may be compounded by the marginal but present inflammatory effect of the SLNs.

Overall, our data suggest that SLNs, whether loaded with mlt or not, do not negatively impact either maternal health or fetal development when administered prior to conception. These findings add additional information to the growing body of evidence supporting the use of lipid-based nanocarriers for drug delivery during pregnancy, indicating their safety also when repeated administration occurs prior to pregnancy.

## Data Availability

The raw data supporting the conclusions of this article will be made available by the authors, without undue reservation.
